# Progress in the Study of B-Cell Immune Metabolic Regulation Mechanisms in Exercise-Induced Fatigue: A Narrative Review

**DOI:** 10.3390/metabo16100752

**Published:** 2026-10-08

**Authors:** Li Yi, Huier Xiang, Guolin Gan, Yixuan Wang, Yi Ruan, Shangjin Song, Wei Gu, Xin Wang

**Affiliations:** 1 Naval Medical Center, Shanghai 200052, China; yl10120901@163.com (L.Y.); xianghuier1998@163.com (H.X.);; 2 Department of Traditional Chinese Medicine, Naval Medical University, Shanghai 200433, China

**Keywords:** exercise-induced fatigue, B lymphocytes, immune metabolism, TGF-β1, glutamate, liver–muscle axis

## Abstract

**Highlights:**

**What are the main findings?**

- This review systematically summarizes the regulatory roles of innate and adaptive immune cells in exercise-induced fatigue, and identifies the B-cell-mediated TGF-β1–liver–muscle glutamate axis as a key cross-organ immune–metabolic regulatory pathway.
- It elaborates the multi-system mediating effects of glutamate, the cross-organ communication between skeletal muscle and immunity, and the putative mechanisms underlying B-cell-related central fatigue.
- Intervention strategies targeting this pathway are framed at three levels: downstream glutamate metabolic compensation, upstream B cell function modulation, and natural product-based comprehensive interventions.

**What are the implications of the main findings?**

- This work expands the classical view of exercise-induced fatigue beyond peripheral energy depletion, providing a novel immune–metabolic perspective for understanding the pathogenesis of exercise fatigue.
- Current research limitations and targeted future directions are clarified, offering guidance for subsequent human cohort validation and development of multi-pronged anti-fatigue strategies.

**Abstract:**

Exercise-induced fatigue refers to the reversible decline in neuromuscular function during physical activity, whose immune–metabolic regulatory mechanisms remain incompletely understood. Recent studies have revealed that B cells can regulate hepatic glutamate metabolism via secretion of transforming growth factor-β1 (TGF-β1), thereby influencing mitochondrial function in skeletal muscle, revealing a novel immune–metabolic pathway relevant to exercise-induced fatigue. This review synthesizes current evidence on immune regulation in exercise-induced fatigue, centering on the TGF-β1-driven glutamate axis linking the liver and skeletal muscle, cross-organ signaling, and hypotheses of central fatigue. We also discuss targeted interventions along this pathway to inform future basic research and clinical management of exercise-induced fatigue.

## 1. Introduction

Exercise-induced fatigue is defined as the reversible impairment of neuromuscular function during physical exertion, which can be classified into central fatigue and peripheral fatigue based on its underlying mechanisms [1]. It is distinct from exercise capacity (maximal performance baseline), muscle injury (structural myofiber damage), recovery (post-exertion functional restoration), and chronic fatigue (persistent pathological state). This review focuses primarily on peripheral fatigue, with a dedicated section covering the putative role of the B-cell–glutamate axis in central fatigue, and the two are clearly delineated throughout the text. The classical view attributes fatigue primarily to depletion of energy substrates and accumulation of metabolic byproducts, with most research focusing on peripheral skeletal muscle processes. However, these models fail to fully explain abnormalities in central nervous regulation or fatigue mediated by immune inflammation. Disruptions in central neurotransmitters and systemic inflammatory responses triggered by exercise are also involved in fatigue development. Cytokines, ammonia, and other immune-related molecules have been shown to participate in the regulation of central fatigue [2]. Furthermore, evidence indicates that regular exercise exerts broad immunomodulatory and anti-inflammatory effects through myokine secretion, remodeling of innate immune cells, and activation of neuroendocrine pathways, serving as a key mechanism for maintaining immune homeostasis [3].

At the level of adaptive immunity, regulatory T cells play a crucial role. Endurance exercise induces expansion of regulatory T cells in muscle tissue, protecting mitochondrial function by suppressing excessive interferon-γ (IFN-γ) production; conversely, deficiency in regulatory T cells directly leads to reduced exercise capacity [4]. Acute high-intensity exercise rapidly promotes migration and infiltration of circulating neutrophils into skeletal muscle, indicating that rapid immune responses are an intrinsic component of the body’s adaptation to exercise stress [5].

Previous research on B cells and exercise fatigue has largely focused on descriptive observations regarding changes in circulating B-cell counts and immunoglobulin levels following exercise. Systematic investigations into whether B cells can directly modulate exercise performance through antibody-independent mechanisms, such as secretion of metabolic factors, have remained limited [6]. In recent years, however, growing evidence suggests that B cells contribute to systemic metabolic homeostasis via cytokine secretion, shedding light on a previously unrecognized link between immune cells and exercise-induced fatigue. A recent study by Mao et al. [7] demonstrated that B cells regulate hepatic glutamate metabolism through secretion of TGF-β1, thereby affecting skeletal muscle function and establishing a B-cell–TGF-β1–liver–muscle glutamate axis. However, existing reviews on exercise-induced fatigue have largely centered on innate immune cells and T-cell-mediated regulation, while the non-canonical, antibody-independent metabolic regulatory functions of B cells have rarely been specifically addressed. This review aims to fill this gap and provide a novel immunometabolic perspective for research in this field. Here we review immune regulatory mechanisms in exercise-induced fatigue, with particular emphasis on the molecular basis of the B-cell–TGF-β1–liver–muscle glutamate axis, cross-talk between skeletal muscle and immunity, and putative central immune pathways. We also examine glutamate as a key mediator and evaluate corresponding therapeutic strategies, with the goal of supporting translational development of anti-fatigue approaches.

For this narrative review, relevant studies were searched in PubMed, Web of Science, CNKI, Wanfang Database and CBM, covering all relevant studies published up to June 2026. Search terms included combinations of “exercise-induced fatigue”, “B lymphocyte”, “B cell”, “immune metabolism”, “transforming growth factor-β1”, “glutamate”, and “liver-muscle axis”. Original research articles and authoritative reviews published in peer-reviewed journals that directly explored immunometabolic regulatory mechanisms in exercise-induced fatigue were included in the analysis. The included studies mainly cover two classic exercise-induced fatigue mouse models, weight-loaded swimming and exhaustive treadmill running, while also incorporating human endurance exercise trials. Differences in conclusions across different models and their extrapolation limitations are addressed in this review. Conference abstracts, case reports, and duplicate records were excluded. This work is presented as a narrative review that integrates and interprets findings from published literature.

## 2. Immune Regulation of Exercise-Induced Fatigue: Foundational Context for B-Cell Immunometabolic Pathways

The onset of exercise-induced fatigue involves not only skeletal muscle energy metabolic disturbances but also multidimensional immune regulation. As foundational context for the B-cell-centered immunometabolic pathway discussed later, this section characterizes established roles of innate immune cells and T lymphocytes in exercise fatigue. Exercise-induced muscle microdamage triggers sequential infiltration of neutrophils, macrophages, and T cells, forming a temporally regulated immune cascade that mediates debris clearance, inflammation modulation, and tissue repair via cytokine and growth factor secretion [8].

### 2.1. Regulatory Roles of Innate Immune Cells

Neutrophils are the first immune cells recruited to the site of exercise-induced muscle injury. Park et al. [5] demonstrated in mouse models that four weeks of treadmill training promoted maturation of circulating neutrophils, which were then capable of effectively enhancing wound healing of skeletal muscle cells in vitro. Neubauer et al. [9] found via transcriptomic analysis that three hours after acute endurance exercise, gene sets related to tissue damage recognition, interleukin-1 receptor, and Toll-like receptor pathways were upregulated in circulating neutrophils. Plasma myoglobin levels correlated positively with neutrophil Toll-like receptor 4(TLR4) gene expression, suggesting that neutrophils can recognize damage-associated molecular patterns released from injured skeletal muscle and initiate transcriptional reprogramming. This pro-inflammatory response is self-limiting; by 48 h post-exercise, neutrophils largely return to a resting state, preventing excessive immune activation. Furthermore, after high-intensity eccentric exercise, plasma interleukin-6 concentrations correlated positively with both neutrophil count and serum creatine kinase (CK) activity, indicating that interleukin-6 may be involved in neutrophil mobilization and subsequent muscle damage progression [10].

Following the initial neutrophil response, macrophages gradually become the primary regulatory cells in muscle repair. Exercise training bidirectionally modulates the polarization of skeletal muscle macrophages: long-term regular training promotes their conversion toward an anti-inflammatory M2 phenotype, maintaining a reparative activated state that supports myofiber regeneration and extracellular matrix remodeling [11]. Castrogiovanni et al. [8] observed age- and sex-related differences in muscle immune responses following resistance exercise: in young females, genes associated with dendritic cells, M1/M2 macrophages, and neutrophils were all upregulated; in young males, activation was primarily seen in M1/M2 macrophage and motor neuron-related genes, whereas in older individuals, activation of M1-type macrophages dominated. Recent studies have shown that high-intensity physical training increases the release of neutrophil extracellular traps (NETs) in athletes [12]. Circulating factors induced by resistance exercise can alter the proteome profile of damaged skeletal muscle within 24 h post-exercise in a sex-dependent manner, with male subjects showing suppression of acute inflammatory pathways, NET formation, and reactive oxygen species production [13]. Nanopeptide scaffolds enhance tendon repair via macrophage-regulated angiogenesis [14].

### 2.2. Regulatory Roles of T Lymphocytes

T lymphocytes play a crucial regulatory role in exercise-induced muscle adaptation. Studies show that after acute skeletal muscle injury, Foxp3^+^ regulatory T cells rapidly accumulate at the injury site, transitioning from a pro-inflammatory to a pro-repair phenotype, directly promoting satellite cell proliferation and tissue regeneration [15]. Langston et al. [4] used conditional knockout mice lacking regulatory T cells and found that these cells increased rapidly in skeletal muscle after exercise. The absence of regulatory T cells led to elevated numbers of interferon-γ-producing cells in muscle, whose overactivation caused mitochondrial swelling, structural disruption, and loss of electron transport chain complexes. Neutralizing interferon-γ with antibodies or specifically knocking out the interferon-γ receptor on muscle fibers can improve motor function and restore mitochondrial function in model mice. The homeostasis of muscle-resident regulatory T cells depends on the interleukin-6 receptor α signaling pathway, a key target mediating interactions between muscle tissue and regulatory T cells; when this signaling is absent, regulatory T cells fail to effectively support muscle functional repair and regeneration [16].

In addition to regulatory T cells, other T-cell subsets also participate in exercise adaptation. Boßlau et al. [17] demonstrated through a randomized controlled trial that 12 weeks of combined strength-endurance training could delay the differentiation of CD8+ T cells into terminal effector memory T cells in older adults, while modulating the activity of the kynurenine metabolic pathway. Baker et al. [18] found that as little as 20 min of incremental-load exercise was sufficient to mobilize γδT cells with cytotoxic, adhesive, and migratory functions into the peripheral circulation. This effect is mediated by β2-adrenergic receptors, suggesting that exercise rapidly modulates the functional state of effector T cells via neuroimmune pathways.

### 2.3. Immune Metabolic Reprogramming: The Metabolic Basis for Immune Cell Functional Switching

Notably, immune cell functional transitions rely on intrinsic metabolic reprogramming. M1 macrophages primarily depend on glycolysis for energy production, whereas M2 macrophages shift toward fatty acid oxidation. Similarly, T-cell activation involves a metabolic switch from oxidative phosphorylation to aerobic glycolysis [19]. This metabolic plasticity represents a critical dimension in understanding how immune cells regulate exercise-induced fatigue. Under exercise stress, immune cells rapidly adjust their energy metabolism to switch from pro-inflammatory to anti-inflammatory functions, thereby precisely controlling the temporal dynamics of muscle damage and repair. B cells also exhibit high metabolic plasticity, with their activation, differentiation, and effector functions strictly regulated by metabolic status [20], laying the foundation for subsequent discussion of how B cells modulate exercise capacity through secretion of metabolic factors.

Collectively, these findings establish immune–metabolic reprogramming as a conserved mechanism governing immune cell function in exercise fatigue. These subsets play distinct and complementary roles: neutrophils and macrophages mediate local damage repair and inflammation resolution; T cells modulate muscle metabolic adaptation and mitochondrial homeostasis. Building on this framework, the following section details an independent systemic immunometabolic axis mediated by B cells via TGF-β1-driven cross-organ glutamate regulation.

## 3. B-Cell-Mediated Immune–Metabolic Regulation of Exercise-Induced Fatigue

While research on T cells has advanced rapidly, the role of B cells in exercise adaptation has long been insufficiently explored. A systematic review by Walzik et al. [6] showed that although exercise alters circulating B-cell counts and immunoglobulin levels, existing studies have not yet established a clear regulatory effect of exercise on classical antibody-mediated adaptive immune functions involving B cells. This reflects the limitations of earlier research, which focused almost exclusively on B cells’ antibody-producing function while overlooking their potential involvement in skeletal muscle homeostasis and exercise performance regulation through non-classical mechanisms. Recent work by Mao et al. [7] demonstrated that B cells can modulate hepatic glutamate metabolism by secreting TGF-β1, which circulates to affect skeletal muscle, improving mitochondrial function and alleviating exercise-induced fatigue—thus establishing a complete cross-organ immune–metabolic regulatory axis (Figure 1).

### 3.1. B-Cell–TGF-β1–Liver–Muscle Glutamate Axis: Experimental Evidence and Molecular Mechanisms

#### 3.1.1. Phenotypic Evidence of Reduced Exercise Endurance in B-Cell-Deficient Mice

Mao et al. [7] used two B-cell-deficient mouse models, namely μMT gene knockout and anti-CD20 antibody-mediated B-cell depletion, and observed reduced exercise endurance, grip strength, and spontaneous activity in both models. However, cardiac ultrasound and skeletal muscle capillary density remained normal, ruling out cardiovascular dysfunction as the cause of impaired physical performance. Additionally, these mice exhibited decreased mitochondrial number and disrupted cristae structure in skeletal muscle, along with downregulated expression of electron transport chain complexes and reduced phosphorylation levels of CaMKII and p70S6K, indicating that B-cell deficiency impairs both mitochondrial structure and calcium signaling. Metabolomics analysis of serum and skeletal muscle identified glutamate as a key mediator; after B-cell depletion, glutamate levels were consistently lower in both resting and exercising states. In vivo U-^13^C-glutamine tracing confirmed that impaired conversion of glutamine to glutamate in the liver was the primary cause of reduced peripheral glutamate availability, with no intrinsic metabolic defect detected in skeletal muscle itself. Exogenous supplementation of glutamate restored exercise capacity and reversed metabolic abnormalities in the model mice, providing functional evidence for a causal link between hepatic glutamate metabolism remodeling and exercise phenotypes. Notably, this reduced exercise capacity reflects increased fatigability and supports involvement in peripheral fatigue, but does not directly confirm effects on subjective or central fatigue.

#### 3.1.2. Transcriptional Mechanism of TGF-β1 in Regulating Hepatic Glutamate Synthesis and Export

Mechanistically, B cells regulate hepatic glutamate metabolism via secretion of TGF-β1. Single-cell RNA sequencing of peripheral blood mononuclear cells revealed that circulating TGF-β1 levels declined following B-cell depletion. This factor acts on TGF-β receptors on hepatocyte surfaces, activating the Smad2/3 signaling pathway to transcriptionally upregulate glutaminase 2 (GLS2) and the amino acid transporter SLC7A5 [7]. GLS2 is the rate-limiting enzyme in the mitochondria of hepatocytes responsible for hydrolyzing glutamine into glutamate, while SLC7A5 forms a heterodimeric complex with SLC3A2 (LAT1-4F2hc) localized at the basolateral membrane of hepatocytes, facilitating the export of newly synthesized glutamate into the bloodstream [21]. Thus, TGF-β1 derived from B cells enhances glutamate output through dual pathways—increased synthesis and enhanced export. Liver-specific knockout of either GLS2 or SLC7A5 resulted in reduced exercise capacity in mice, further confirming the necessity of this pathway.

This regulatory mechanism aligns with previous findings. Studies on liver diseases have shown that B cells can modulate hepatocyte function and tissue repair via non-antibody-dependent paracrine pathways [22]. The liver serves as a central hub in the inter-organ metabolic network of amino acids such as glutamate, glutamine, citrulline, and arginine [23]. The transforming growth factor-β signaling pathway can reshape hepatic cell oxidative metabolism by regulating the expression of SLC7A5 and glutaminase 2 [24]. Notably, B-cell subsets that secrete transforming growth factor-β1 may represent regulatory B cells, which modulate inflammatory responses through the production of anti-inflammatory factors such as interleukin-10 and transforming growth factor-β [25]. However, the specific activation mechanisms of regulatory B cells under exercise conditions and their precise interaction with hepatocyte metabolism remain to be further elucidated.

#### 3.1.3. Glutamate–Ca^2+^/CaMKII–Mitochondrial Biogenesis Axis

Glutamate entering skeletal muscle can induce cytosolic calcium oscillations, activate CaMKII-mediated downstream signaling pathways, promote mitochondrial biogenesis, and ultimately maintain muscle contractile function [7]. Upon activation by calcium signals, CaMKII phosphorylates p38 MAPK, thereby activating transcription factors such as CREB to initiate PGC-1α transcription, synergistically driving mitochondrial protein expression and enhancing oxidative phosphorylation capacity [26]. In the absence of B cells, insufficient glutamate supply to skeletal muscle leads to reduced activity of calcium-signaling downstream pathways, further exacerbating mitochondrial structural damage. Complementing this systemic endocrine regulation, skeletal muscle macrophage subsets can locally release glutamate to activate muscle spindles and regulate motor coordination [27]. These two pathways, operating at both systemic endocrine and local tissue microenvironment levels, demonstrate the broad applicability of immune cell regulation over skeletal muscle glutamate function. Clinically, abnormal B-cell activation in patients with polymyositis and dermatomyositis can mediate muscle damage; rituximab-induced B-cell depletion therapy suppresses aberrant immune responses and improves muscle strength [28], providing independent clinical evidence for B-cell regulation of skeletal muscle function. In support of a pathogenic role for TGF-β signaling in muscle dysfunction, excessive TGF-β activation has been shown to suppress satellite cell proliferation, activation, and differentiation, thereby impairing muscle regeneration. Conversely, inhibition of TGF-β-related pathways can attenuate fibrosis and improve muscle architecture and functional outcomes in experimental myopathy models [29].

### 3.2. Glutamate’s Multisystem Mediator Effects and Regulatory Network

Metabolic studies of exercise-induced fatigue have long centered on classic biochemical markers: lactate, urea, and creatine kinase. Lactate accumulates with enhanced glycolysis during high-intensity exercise and serves as a sensitive indicator of metabolic stress, but its levels are confounded by exercise intensity, training status and recovery time, and it does not directly reflect the regulatory pathways driving fatigue [30]. Urea and blood urea nitrogen reflect increased protein catabolism and nitrogen turnover under exercise load, while CK marks skeletal muscle micro-injury; both have limited specificity for fatigue per se, as they are strongly influenced by diet, renal function and individual variability [31,32].

As a key downstream effector molecule in the B-cell–TGF-β1–liver–muscle glutamate axis, glutamate differs from these descriptive stress markers. It functions not only as an energy substrate for skeletal muscle, but also as an active immunometabolic mediator that bridges immune signaling, hepatic metabolism and skeletal muscle function, and participates in coordinated regulation between central and peripheral systems.

#### 3.2.1. Skeletal Muscle Metabolic Support and Receptor Signaling Pathways

As a non-essential amino acid with critical metabolic functions in skeletal muscle energy metabolism, glutamate performs multiple fundamental metabolic roles, including replenishing the tricarboxylic acid cycle, supplying glutathione precursors, and supporting purine nucleotide cycling [33]. It regulates skeletal muscle metabolic homeostasis through multiple signaling pathways. Long-term aerobic exercise reduces circulating glutamate levels while enhancing the activity of the InsR/IRS1/Akt pathway in skeletal muscle, improving whole-body insulin sensitivity, indicating that its regulatory effects extend beyond mere energy substrate provision [34]. In myotube cells, glutamate promotes glucose uptake via the AMPK pathway in a dose- and time-dependent manner, with efficacy comparable to leucine and insulin [35]. Furthermore, resistance exercise induces persistent remodeling of glutamate metabolism, with glutamate serving as an exercise-responsive metabolite involved in metabolic adaptation and signaling regulation [36]. Insufficient glutamate supply leads to reduced levels of tricarboxylic acid cycle intermediates and diminished ammonia clearance capacity, accelerating metabolic disturbances and inducing muscle fatigue. This represents a key downstream mechanism by which B-cell-regulated pathways influence exercise performance. Skeletal muscle expresses functional glutamate receptors, enabling glutamate to directly modulate muscle function via receptor-mediated signaling pathways. Human primary skeletal muscle cells express the AMPA-type glutamate receptor GluR3 subunit; upon binding glutamate, intracellular sodium concentration increases directly, promoting cell proliferation, indicating that skeletal muscle can respond directly to glutamate signals and undergo functional changes [37]. NMDA-type glutamate receptors are also functionally expressed in skeletal muscle; glutamate binding to these receptors suppresses the activity of the InsR/IRS1/PI3K/Akt pathway within myotubes. This inhibitory effect disappears when the NMDA receptor inhibitor MK-801 is added, demonstrating that glutamate regulates skeletal muscle metabolic reprogramming through receptor-mediated signaling pathways [34].

#### 3.2.2. Central–Peripheral Glutamate Homeostasis and Coordinated Regulation

There exists a glutamate concentration gradient between peripheral blood and the central nervous system, with blood glutamate levels exceeding those in cerebrospinal fluid by more than 50-fold. Maintenance of this homeostasis depends on the integrity of the blood–brain barrier(BBB) and active regulation by endothelial glutamate transporters [38]. Exercise influences both the metabolism and functional status of central glutamate. Yu et al. [39] found that exercise enhances phosphorylation levels of NMDA and AMPA receptors, improving astrocytic glutamate clearance capacity. Human studies have also confirmed elevated cortical glutamate concentrations following acute high-intensity exercise. These findings indicate that exercise modulates the activity of central glutamatergic signaling. The B-cell-regulated transforming growth factor-β1 liver-derived pathway not only maintains peripheral glutamate levels and ensures mitochondrial energy supply in skeletal muscle but also indirectly affects central glutamate homeostasis through changes in peripheral glutamate concentrations, highlighting glutamate’s role as a mediator in the coordinated regulation of immune, metabolic, and neural functions.

### 3.3. B-Cell-Mediated Cross-Organ Communication Between Skeletal Muscle and Immunity

Exercise-induced skeletal muscle contraction releases myokines that initiate systemic immune–metabolic responses, mediating inter-organ communication. Interleukin-6 (IL-6) and interleukin-15 (IL-15) are key endocrine mediators in this process. IL-6 promotes the formation of an anti-inflammatory microenvironment by regulating B-cell differentiation and immunomodulatory functions, contributing to inflammation control and tissue repair after exercise-induced damage [40]. IL-15 modulates the energy metabolism of immune cells and drives functional remodeling of B cells, playing a role in maintaining post-exercise inflammatory homeostasis [41]. Immunometabolic reprogramming of B cells may represent a critical step in translating myogenic signals into immune effector responses. Exercise-induced myokines can alter B-cell nutrient-sensing pathways and metabolic profiles, changing their subset composition and immunoregulatory capacity, thereby influencing the onset and progression of exercise-induced fatigue [19,20].

However, these regulatory mechanisms remain largely theoretical and lack direct in vivo validation under exercise conditions. Cross-organ communication between skeletal muscle and the immune system forms a fundamental basis for systemic adaptation to exercise. Whether B cells act as key immunometabolic sensor cells that receive exercise-derived signals and convert them into adaptive immune responses remains to be further investigated using experimental approaches such as B-cell conditional knockout, myokine neutralization, and metabolic tracing.

### 3.4. B Cells and Central Fatigue: Hypotheses and Potential Mechanisms

Central fatigue is a key contributor to diminished exercise capacity, with the crosstalk between the brain and immune system playing a crucial regulatory role. Currently, research on the direct regulation of central fatigue by B cells during exercise remains largely theoretical, lacking direct experimental evidence. Based on the immunomodulatory functions and metabolic characteristics of B cells, the following working hypothesis can be proposed:

Exercise-induced release of myokines such as interleukin-6 from skeletal muscle exerts distinct immunomodulatory effects compared to those in infectious states, promoting the production of anti-inflammatory factors and suppressing tumor necrosis factor-α (TNF-α)-mediated inflammatory responses, thereby creating a peripheral environment conducive to tissue recovery [40]. During prolonged high-intensity exercise or inadequate recovery, persistent elevation of peripheral pro-inflammatory cytokines may cross the BBB, activate the vagus nerve, or engage endothelial signaling pathways to influence the central nervous system. This leads to abnormal activation of microglia, increased secretion of interleukin-1β (IL-1β), TNF-α, and reactive oxygen species, exacerbating neuroinflammation and impairing neural function [42]. B cells are not only antibody-producing cells but also important immunoregulatory cells; among them, regulatory B cells modulate inflammatory responses by secreting anti-inflammatory cytokines such as interleukin-10 and TGF-β1 [25]. Under central pathological conditions, regulatory B cells can infiltrate the brain parenchyma through a compromised blood–brain barrier, suppress excessive microglial activation, and alleviate neuroinflammation, thus exerting neuroprotective effects [43].

It is hypothesized that during exercise, B cells may receive signals from skeletal muscle-derived myokines, undergo immunometabolic reprogramming, and consequently alter their functional state, indirectly influencing the inflammatory milieu within the brain. The coordinated regulation between the brain and immune system offers a novel perspective for understanding the mechanisms underlying exercise-induced central fatigue. Given their dual capabilities in immune modulation and metabolic remodeling, B cells may serve as a critical node linking muscles, the immune system, and the brain. Future integration of multi-omics technologies—including single-cell sequencing and spatial transcriptomics—could further clarify their precise mechanisms of action.

## 4. Intervention Strategies Targeting B-Cell Immune Metabolic Pathways

The B-cell-mediated liver–muscle glutamate metabolic pathway represents a key target in research on interventions for exercise-induced fatigue. This pathway encompasses critical nodes including hepatic glutamate synthesis and efflux, circulating glutamate levels, and calcium-dependent mitochondrial biogenesis in skeletal muscle—each of which holds potential for therapeutic modulation. Based on this regulatory axis, intervention strategies can be developed at three levels: downstream metabolic compensation, upstream immune regulation, and natural product-based interventions.

### 4.1. Downstream Interventions Targeting Glutamate Metabolism

Glutamine serves as a precursor to glutamate; supplementing exogenous glutamine increases circulating glutamine levels, providing ample substrate for hepatic GLS2 activity and thereby enhancing glutamate production. Systematic reviews in sports nutrition suggest that glutamine exerts anti-fatigue effects through multiple mechanisms, such as promoting glycogen synthesis, reducing ammonia accumulation, and maintaining acid-base balance. However, its impact on athletic performance remains inconsistent across studies [44]. A study simulating hypoxic conditions equivalent to an altitude of 4200 m demonstrated that six consecutive days of glutamine supplementation (20 g/day), combined with carbohydrate intake during exercise, could suppress the rise in subjective fatigue ratings [45]. Regarding strength recovery, glutamine supplementation at 0.3 g per kilogram of body weight reduced post-exercise strength decline and alleviated perceived muscle soreness in recreationally active men following eccentric exercise [46]. Recent evidence indicates that glutamine supports post-exercise recovery by mitigating muscle inflammation, improving redox balance, and enhancing anabolic signaling pathways. Nevertheless, its efficacy is influenced by dosage, timing, and individual variability, and the benefits observed in healthy athletes appear weaker compared to those seen in clinical populations under high physiological stress [47].

From the perspective of the B-cell–TGF-β1–liver–muscle glutamate axis, glutamine supplementation bypasses upstream B-cell regulation and directly enhances hepatic glutamate synthesis, compensating for insufficient production due to downregulated GLS2 expression. This constitutes a compensatory downstream intervention strategy. It may be effective when B-cell function is temporarily impaired or when glutamate supply is inadequate due to excessive exercise stress, but it does not restore the underlying immune–metabolic regulatory capacity of B cells. Additionally, direct glutamate supplementation or activation of the CaMKII pathway may serve as alternative options for rapidly improving exercise performance, although their long-term safety and optimal dosing require further validation.

### 4.2. Upstream Interventions Targeting B-Cell Function

Restoring B-cell immune regulatory function at the upstream level may offer a more precise intervention approach. During exercise training adaptation, immune cells participate in skeletal muscle repair by coordinately regulating inflammatory responses and metabolic homeostasis, highlighting the potential of targeted immune modulation to enhance athletic performance [48]. Studies show that regular physical activity reshapes B-cell subset composition, promotes expansion of regulatory B cells, and enhances their ability to secrete anti-inflammatory factors such as TGF-β1 [6,25]. Therefore, scientifically designed training programs including controlled exercise intensity and adequate recovery periods can themselves serve as non-pharmacological interventions to optimize B-cell function. Furthermore, precise modulation of the TGF-β1 signaling pathway presents another promising avenue, such as using small-molecule agonists to increase hepatocyte sensitivity to TGF-β receptors or employing epigenetic approaches to enhance transcriptional efficiency of GLS2 and SLC7A5. However, direct evidence in exercise contexts remains limited.

The gut microbiota–immune axis represents another promising upstream target. Metabolites produced by gut microbes, such as short-chain fatty acids, regulate B-cell differentiation and metabolic status, thereby influencing systemic immune homeostasis. Exercise itself can alter the composition of the gut microbiota, suggesting a more complex regulatory network whose underlying mechanisms require further clarification through integrative multi-omics research.

### 4.3. Natural Products and Comprehensive Interventions

In studies on natural products, certain compounds may indirectly affect B-cell function or glutamate metabolism by modulating inflammatory and metabolic pathways. For example, diosgenin has been shown to improve oxidative stress and TNF-α-mediated inflammatory responses in chronic fatigue syndrome models, thereby restoring exercise capacity in animals [49]. Similarly, the herbal formula Shenqi Funeng Xingnao prescription can ameliorate dysfunctions in gastrointestinal–central nervous system interactions by regulating the TNF/NOD-like receptor pathway, alleviating exercise-induced central fatigue [50]. Although these studies have not directly validated effects on the B-cell–TGF-β1–liver–muscle glutamate axis, their anti-inflammatory and metabolic regulatory effects may intersect with this pathway. Future research could screen and validate natural active components that directly target B-cell metabolic reprogramming or specifically modulate TGF-β1, GLS2, and SLC7A5 signaling pathways, thus advancing the development of precision-based functional foods and sports nutrition supplements for fatigue prevention.

## 5. Conclusions

Exercise-induced fatigue results from coordinated regulation across multiple systems, with immune–metabolic crosstalk playing a crucial role in its onset and recovery. Collectively, the evidence summarized here positions the TGF-β1-mediated glutamate axis linking B cells, the liver, and skeletal muscle as a key node regulating exercise capacity and peripheral fatigue in exercise-induced fatigue. This pathway confirms that B cells possess non-classical metabolic regulatory functions beyond antibody production. Specifically, they can secrete TGF-β1 to regulate hepatic glutamate output, thereby influencing mitochondrial biogenesis and contractile function in skeletal muscle. This framework expands the canonical view of exercise fatigue beyond peripheral energy depletion to include immune–metabolic cross-organ signaling. Notably, current evidence primarily supports the role of this axis in regulating peripheral fatigue, while its involvement in central fatigue remains largely inferential and requires further validation. Targeting nodes along this pathway, such as glutamine and glutamate supplementation, immune modulation, and gut microbiota interventions, creates opportunities for multi-pronged anti-fatigue strategies.

However, current research still faces significant limitations. First, evidence supporting the B-cell-regulated liver–muscle glutamate pathway primarily comes from rodent models, mainly involving two classic exercise paradigms: weight-loaded swimming and exhaustive treadmill running. Human validation data remain insufficient, and the activation patterns and dose–response relationships of this pathway under different exercise modes (acute exhaustive exercise vs. chronic endurance training) and among diverse populations remain unclear, limiting the extrapolation of current findings. Second, inter-organ communication between skeletal muscle and the immune system, as well as central immune interactions, are largely based on theoretical hypotheses, lacking direct experimental evidence. Third, existing studies exhibit notable gender bias, with most mechanistic evidence derived from male animals and male human subjects. Given sex differences in sex hormone levels, immune cell composition, and metabolic regulation, the responsiveness of the B-cell–TGF-β1–glutamate axis may be sex-dependent, which remains to be systematically investigated. Fourth, the clinical translation potential of existing intervention strategies remains inadequately demonstrated, particularly upstream interventions targeting B-cell function, which are still in the conceptual stage.

Future research should focus on several directions. First, human cohort studies combining exercise load tests with metabolomics and single-cell sequencing technologies should be conducted to validate the activation patterns of the B-cell–TGF-β1–liver–muscle axis in human exercise-induced fatigue. Second, techniques such as B-cell conditional knockout, optogenetics, and metabolic tracing should be employed to clarify the specific response mechanisms of distinct B-cell subsets—especially regulatory B cells—during exercise stress. Third, integrating spatial transcriptomics with multi-omics analysis will help elucidate the nodal functions of B cells within the muscle–liver–brain inter-organ communication network. Fourth, natural active molecules or sports nutrition supplements targeting the TGF-β1, GLS2, and SLC7A5 pathways should be screened to accelerate the clinical translation of therapeutic targets. In-depth exploration along these lines will further strengthen the experimental foundation for precise prevention and treatment of exercise-induced fatigue.

## Figures and Tables

**Figure 1:**
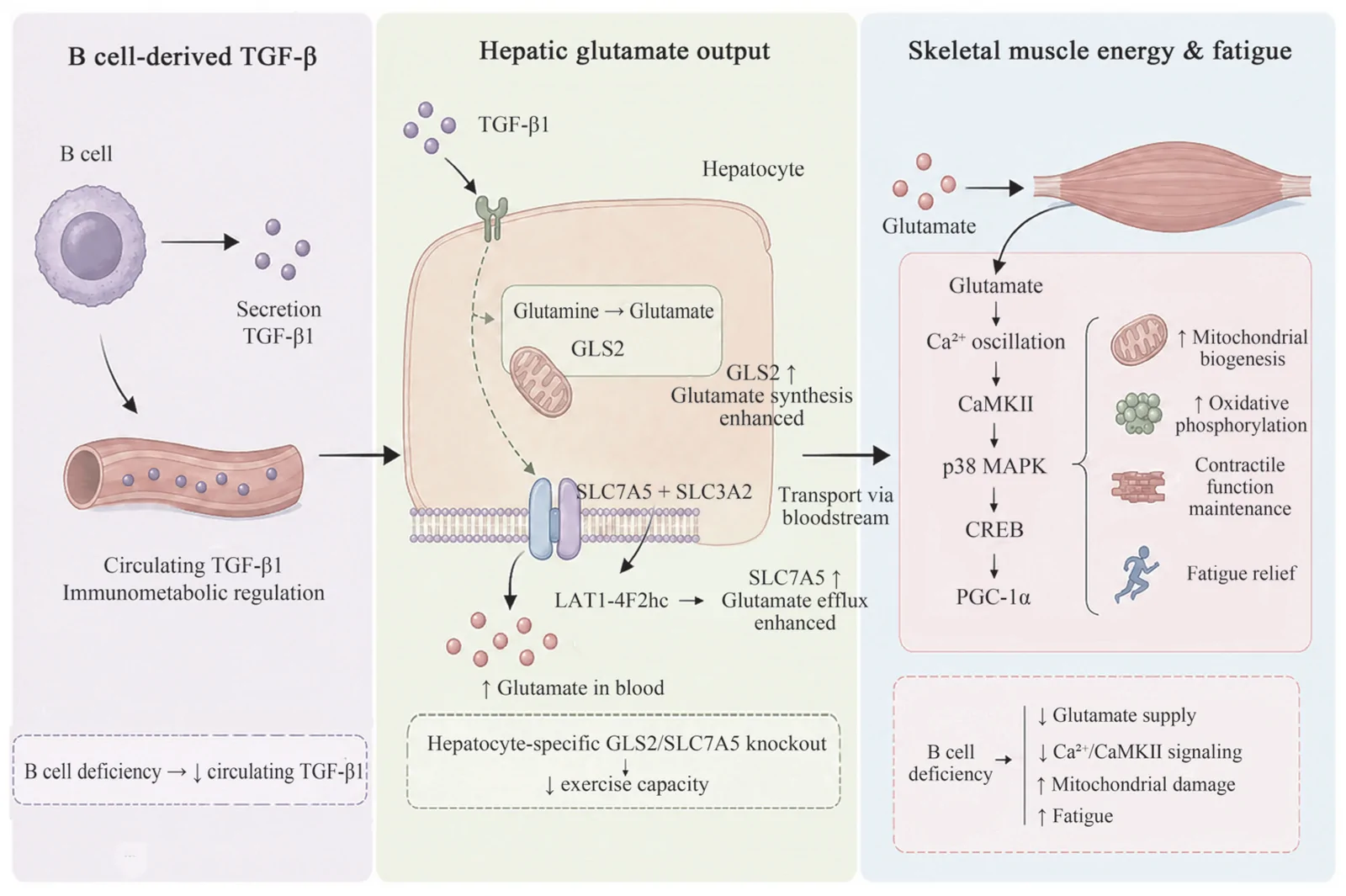
B-cell–TGF-β1–liver–muscle glutamate axis in exercise-induced fatigue-regulation B cells secretes TGF-β1, which acts on hepatocytes to transcriptionally upregulate GLS2 and the amino acid transporter SLC7A5, thereby promoting glutamate synthesis and release in the liver. Glutamate enters skeletal muscle via the bloodstream, activates the Ca^2+^/CaMKII signaling pathway, and stimulates mitochondrial biogenesis, thus maintaining muscle contractile function and alleviating exercise-induced fatigue. Conversely, B-cell deficiency or liver-specific knockout of these key genes disrupts this metabolic axis, leading to insufficient glutamate supply and mitochondrial structural damage, thereby exacerbating exercise-induced fatigue. Upward arrows indicate upregulated expression or enhanced activity of corresponding molecules and pathways, while downward arrows indicate downregulated expression or impaired function.

## Data Availability

No new data were created or analyzed in this study. Data sharing is not applicable to this article.

## Abbreviations

The following abbreviations are used in this manuscript:

AMPK	Adenosine monophosphate-activated protein kinase
AMPA	α-amino-3-hydroxy-5-methyl-4-isoxazolepropionic acid
Akt	Protein kinase B
BBB	Blood–brain barrier
CaMKII	Calcium/calmodulin-dependent protein kinase II
CD20	Cluster of differentiation 20
CD8	Cluster of differentiation 8
CK	Creatine kinase
CREB	cAMP response element-binding protein
Foxp3	Forkhead box protein 3
GLS2	Glutaminase 2
IFN-γ	Interferon-γ
IL-1β	Interleukin-1β
IL-6	Interleukin-6
IL-10	Interleukin-10
IL-15	Interleukin-15
InsR	Insulin receptor
IRS1	Insulin receptor substrate 1
LAT1-4F2hc	L-type amino acid transporter 1-4F2 heavy chain
MAPK	Mitogen-activated protein kinase
μMT	μ heavy chain-deficient mouse
NETs	Neutrophil extracellular traps
NMDA	N-methyl-D-aspartate
PGC-1α	Peroxisome proliferator-activated receptor gamma coactivator 1-alpha
PI3K	Phosphoinositide 3-kinase
p70S6K	p70 ribosomal S6 kinase
Smad2/3	Mothers against decapentaplegic homolog 2/3
SLC3A2	Solute carrier family 3 member 2
SLC7A5	Solute carrier family 7 member 5
TGF-β1	Transforming growth factor-β1
TLR4	Toll-like receptor 4
TNF-α	Tumor necrosis factor-α
